# Alpha-1 microglobulin as a potential therapeutic candidate for treatment of hypertension and oxidative stress in the STOX1 preeclampsia mouse model

**DOI:** 10.1038/s41598-019-44639-9

**Published:** 2019-06-12

**Authors:** Lena Erlandsson, Aurélien Ducat, Johann Castille, Isac Zia, Grigorios Kalapotharakos, Erik Hedström, Jean-Luc Vilotte, Daniel Vaiman, Stefan R. Hansson

**Affiliations:** 1 Obstetrics and Gynecology, Department of Clinical Sciences Lund, Lund University, Lund, Sweden; 2 INSERM U1016, CNRS UMR8104, Faculté de Médecine, Institut Cochin, Paris, France; 3 INRA-AgroParisTech, UMR1313 Génétique Animale et Biologie Intégrative, Institut National de la Recherche Agronomique, Jouy-en-Josas, France; 4 Clinical Physiology, Department of Clinical Sciences Lund, Lund University, Lund, Sweden; 5 Diagnostic Radiology, Department of Clinical Sciences Lund, Lund University, Lund, Sweden

**Keywords:** Preclinical research, Molecular medicine

## Abstract

Preeclampsia is a human placental disorder affecting 2–8% of pregnancies worldwide annually, with hypertension and proteinuria appearing after 20 weeks of gestation. The underlying cause is believed to be incomplete trophoblast invasion of the maternal spiral arteries during placentation in the first trimester, resulting in oxidative and nitrative stress as well as maternal inflammation and organ alterations. In the Storkhead box 1 (STOX1) preeclampsia mouse model, pregnant females develop severe and early onset manifestations as seen in human preeclampsia e.g. gestational hypertension, proteinuria, and organ alterations. Here we aimed to evaluate the therapeutic potential of human recombinant alpha-1 microglobulin (rA1M) to alleviate the manifestations observed. Human rA1M significantly reduced the hypertension during gestation and significantly reduced the level of hypoxia and nitrative stress in the placenta. In addition, rA1M treatment reduced cellular damage in both placenta and kidneys, thereby protecting the tissue and improving their function. This study confirms that rA1M has the potential as a therapeutic drug in preeclampsia, and likely also in other pathological conditions associated with oxidative stress, by preserving normal organ function.

## Introduction

Preeclampsia is a human placental disorder that clinically presents after 20 weeks of gestation with maternal manifestations including hypertension and proteinuria^1,2^. Around 2–8% of pregnancies worldwide are affected annually^3^. The placenta plays a key role in the development of the disease, as its removal results in resolution of the clinical signs but also contributes to the high rate of premature births. Even if the maternal manifestations appear in the third trimester, the underlying cause is believed to be incomplete trophoblast differentiation/invasion of the maternal spiral arteries during placentation in the first trimester. Defective remodeling of the maternal spiral arteries is believed to result in high pressure flow entering the intervillous space, causing physical disruption of the placental villous architecture and fluctuations in oxygen delivery with relative hypoxia in the placenta^4^. This leads to increased inflammation^5,6^, oxidative stress^7,8^, and nitrative stress^9,10^. In addition, oxidative stress is closely linked to endoplasmic reticulum (ER) stress and redox homeostasis^11,12^. Normal pregnancy is in a state of oxidative stress compared to the non-pregnant state, generating reactive oxygen species (ROS)^13,14^, which is further elevated in complicated pregnancies such as preeclampsia^8,15^. The maternal manifestations occurring after 20 weeks of gestation can be viewed as the maternal response towards placenta-derived circulating factors released through a disrupted placental barrier, resulting in systemic endothelial activation and dysfunction^16^. Women suffering from severe preeclampsia can develop eclampsia and seizures. Damage to the heart can cause peripartum cardiomyopathy^17^, postpartum cardiovascular impairment^18^, and severe cases also have increased risk of cardiovascular disease and stroke later in life^19^. Pre-symptomatic low-dose aspirin treatment (before 16 weeks of pregnancy) is efficient to prevent the onset of preeclampsia^20^, but there is a clear lack of pharmaceutical therapeutic solutions able to alleviate the symptoms once they occur, allowing to safely prolong the pregnancy. Therefore such therapeutic approaches are much needed.

In a transgenic mouse model overexpressing the transcription factor Storkhead box 1 (STOX1) gene, pregnant females develop typical human preeclampsia manifestations such as gestational hypertension, proteinuria, kidney and placental tissue alterations, reduced litter size, and increased plasma levels of the anti-angiogenic factors soluble fms-like tyrosine kinase 1 (s-Flt1) and soluble endoglin (sEng)^21^. By mating wildtype (wt) females with STOX1 transgenic males, the transgene expression is restricted to the fetoplacental unit, making this one of the few animal models representing a severe and early onset form of preeclampsia. Furthermore, these pregnant females display cardiac hypertrophy and endothelial cell deregulation in gene networks linked to oxidative stress, cell cycle, and hypertrophy^22^. In addition, the placentas show alterations in mitochondria-related pathways and a disrupted nitroso-redox balance^23^. The STOX1 overexpression also results in hyperactive mitochondria, which leads to increased free radical production. In addition, nitric oxide (NO) production pathways are activated, generating peroxynitrite as a result of NO reacting with superoxide. Peroxynitrite is highly unstable and reacts with tyrosine residues on proteins, producing nitrotyrosine (protein nitration)^24^. Protein nitration has been shown to occur in a number of pathological conditions associated with inflammation, including preeclampsia^25,26^. Elevated levels of ROS that reacts with NO would result in decreased levels of NO, which is an important vasodilator in the vascular system, thereby potentially contributing to the hypertension described in this mouse model.

The endogenous protein alpha-1-microglobulin (A1M) is a ~30 kDa protein with haem- and radical-binding capacity as well as reductase activity. It has been shown to be protective against oxidative stress, as well as an inducer of natural tissue repair mechanisms^27^. Mainly synthesized by the liver, A1M is present both in the circulation as well as in the extravascular compartments. In the circulation, it forms complexes with other proteins in particular prothrombin. It is re-cycled in the kidneys and if present in urine, it is a major marker of renal tubular function^28^. It has also been shown to prevent intracellular oxidation, protect mitochondria, prevent mitochondrial swelling, inhibit cell lysis, and repair lesions caused by oxidative stress (reviewed in^27^). Exogenously administered recombinant A1M (rA1M) has previously been shown to have a therapeutic effect against organ damage induced by cell-free haemoglobin in organs such as placenta and kidneys, both *ex vivo*^29^ and *in vivo* in other less specific preeclampsia animal models^30,31^.

Here we used the STOX1 mouse model of severe preeclampsia to explore in detail the therapeutic possibilities of rA1M treatment in preeclampsia. The STOX1 transgene mouse model provides a useful model for analysing in depth and in an organ-targeted way the pathophysiological consequences of preeclampsia. It also offers the opportunity to investigate ways of reducing oxidative stress in preeclampsia, as well as testing new therapeutic avenues.

## Results

### Human rA1M significantly alleviates hypertension during mid- and late gestation

**Figure 1:**
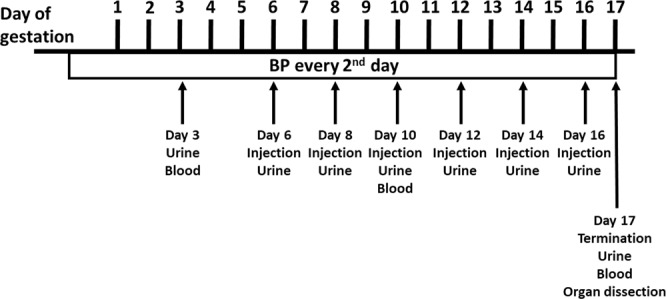
Experimental design. Illustration of the experimental design, indicating time points for collection of urine and blood, blood pressure measurements, injections and terminations.

**Figure 2:**
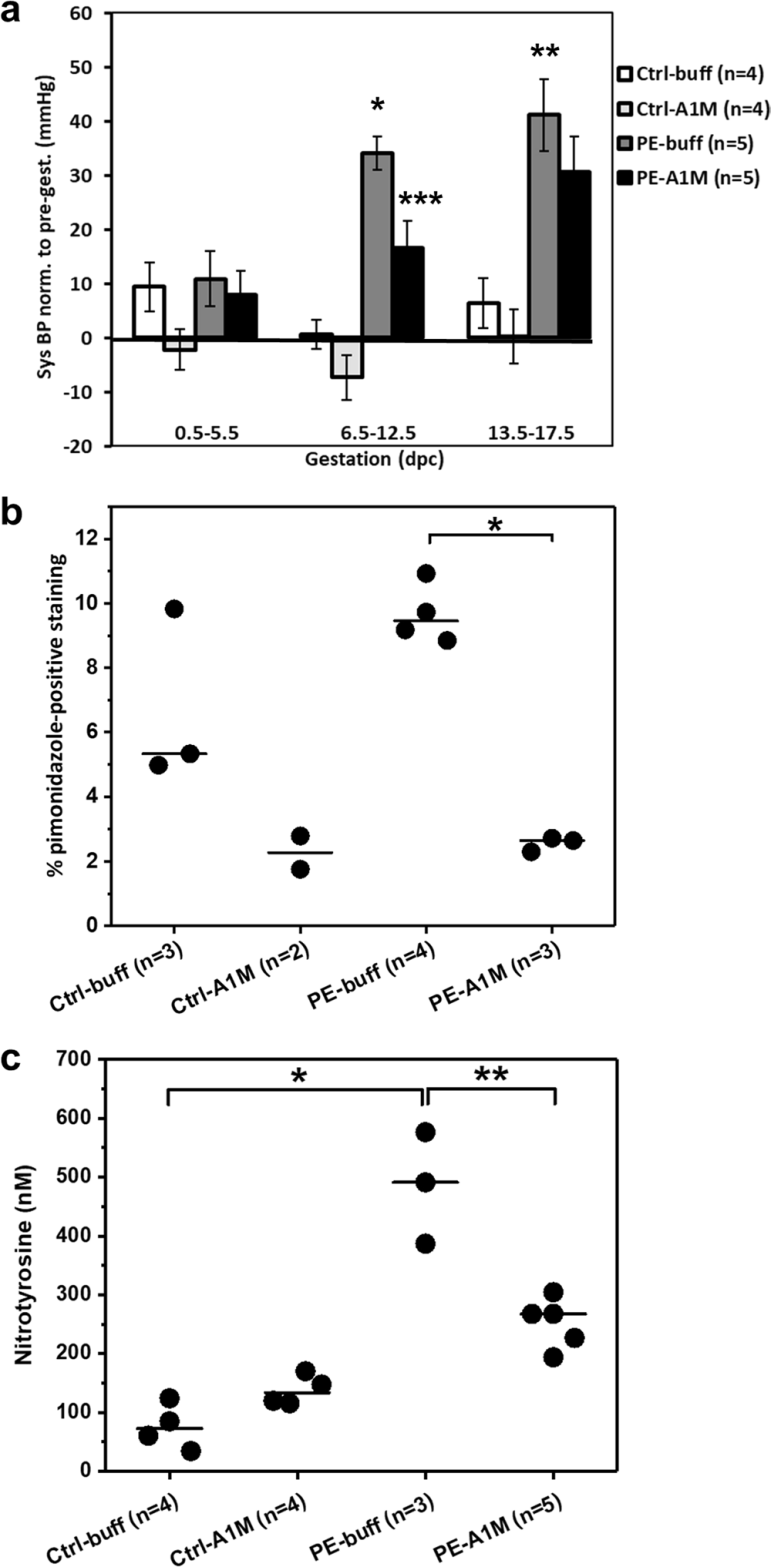
Human rA1M significantly reduces hypertension and placental hypoxia/nitrative stress levels in preeclamptic females. (**a**) Systolic BP measurements during early-, mid- and late pregnancy, normalised to pre-gestation pressure (mmHg). The PE-buff group displayed significantly elevated BP at mid and late gestation, compared to Ctrl-buff (*p = 5 × 10^−9^, **p = 5 × 10^−4^). Human rA1M significantly reduced BP mid-gestation, compared to PE-buff group (***p = 0.007). Shown is mean ± SEM, with 10–18 BP measurements for each gestation period and group. (**b**) Hypoxyprobe immunohistochemistry demonstrated a trend of higher levels of hypoxia in the junctional zone of preeclamptic placentas at 17.5 dpc, compared to controls. This was significantly reduced by rA1M treatment (PE-A1M vs PE-buff, *p < 0.0001). The line represent the median, and n = number of females analysed with three-four placentas/female. (**c**) Significantly elevated levels of protein nitration in the preeclamptic placentas at 17.5 dpc compared to controls (*p = 0.05), which was significantly reduced by rA1M treatment (**p = 0.04). The line represents median, and n = number of females analysed with one placenta/female.

The experimental set-up is described in Fig. 1. Females were given six i.p. injections of either buffer or rA1M every second day starting at 6.5 dpc. Human rA1M was detected in plasma at timepoint 10.5 dpc from rA1M-treated females, confirming that i.p. injected rA1M reached the circulation (Supplementary Fig. S1). Blood pressure (BP) was measured throughout gestation and the preeclamptic females (PE-buff) showed a significant increase in systolic BP during both mid- (p = 5 × 10^−9^) and late-gestation (p = 5 × 10^−4^) when compared to control groups (Fig. 2a and Supplementary Fig. S2). This increase was significantly alleviated by rA1M treatment during mid-gestation compared to PE-buff group (p = 0.007) and to some extent also during late-gestation. There was no increase in BP during gestation in the control groups.

### Human rA1M improves placental weight

Preeclamptic females showed a tendency towards reduced litter size at 17.5 days post coitum (dpc) compared to controls (Table 1), which was not affected by rA1M treatment. Also, preeclamptic females demonstrated significantly reduced placental weight compared to controls (p = 0.0001), which was alleviated in the PE-A1M group (Table 2). There was no significant difference in foetal weight at day 17.5 dpc between any of the groups.

**Table 1:** Litter size.

Groups	Ctrl-buff (n = 4)	Ctrl-A1M (n = 4)	PE-buff (n = 5)	PE-A1M (n = 5)
pups/litter	8 (4–9)	7 (6–8)	5 (1–8)	5 (2–11)

Shown is median (range) at time of termination (17.5 dpc). N = number of females.

**Table 2:** Placental and foetal weight at 17.5 dpc.

Groups	Placental weight (mg)	Foetal weight (mg)
Ctrl-buff (n = 4)	69 (55–114)	841 (679–955)
Ctrl-A1M (n = 2)	66 (56–88)	845 (736–1188)
PE-buff (n = 6)	61 (46–79)*	856 (619–1117)
PE-A1M (n = 3)	66 (40–96)	883 (605–1074)

Shown is median (range) at time of termination (17.5 dpc). Mann-Whitney U-test: PE-buff vs Ctrl-buff *p = 0.0001. N = number of females.

### Human rA1M reduces the level of hypoxia and nitrative stress in the preeclamptic placenta

We used Hypoxyprobe^TM^ immunohistochemistry to identify hypoxic regions in the mouse placenta at gestational age 17.5 dpc (Supplementary Fig. S3). The majority of Hypoxyprobe staining was in the junctional zone of the placenta, and there was a trend of higher levels of hypoxia in the preeclamptic placentas compared to controls (p = 0.06), which was significantly reduced by rA1M treatment (p = 1 × 10^−6^) (Fig. 2b). Furthermore, the preeclamptic mouse placentas showed significantly elevated levels of protein nitration when compared to controls (p = 0.05) (Fig. 2c). Human rA1M treatment resulted in significantly lower levels in the rA1M-treated preeclampsia group compared to PE-buff (p = 0.04).

### Human rA1M protects the placental tissue

**Figure 3:**
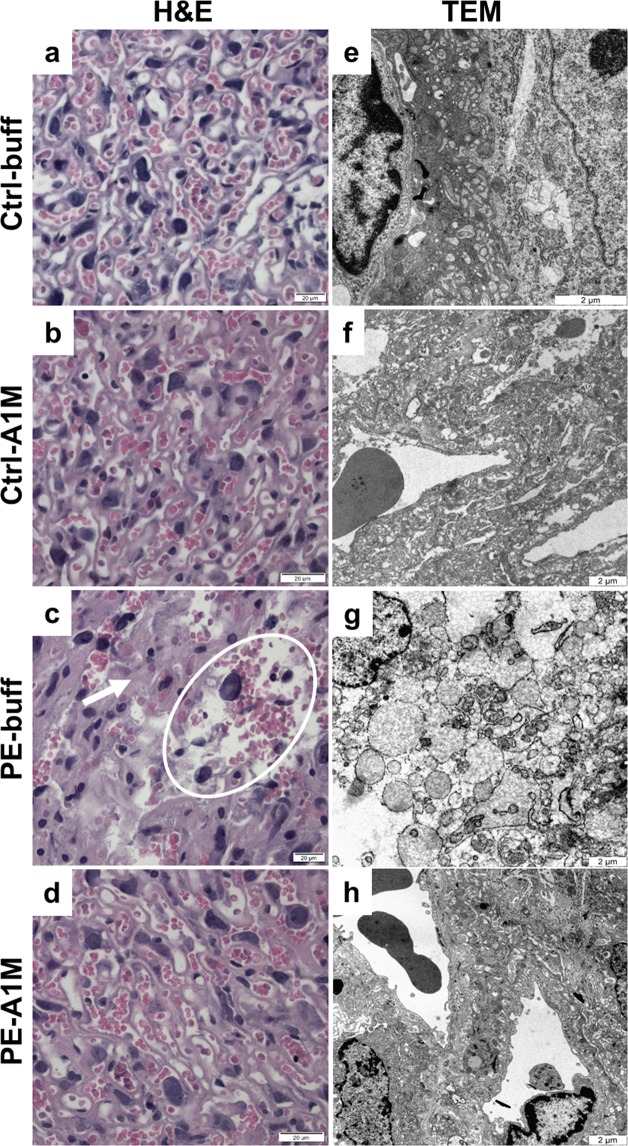
Histological and TEM analyses of placenta tissue structure. Morphological analyses of placenta biopsies at 17.5 dpc using H&E staining (**a-d**, scale bar = 20 µm) and TEM analysis (**e**–**h**, scale bar = 2 µm), showing representative images. (**a,b**) Control groups displayed normal tissue morphology. (**c**) The labyrinth zone of preeclamptic placentas displayed areas of necrosis (circle) and swollen tissue (arrow). (**d**) Placentas from rA1M-treated preeclamptic females showed no swelling and no necrosis, and were indistinguishable from control groups. (**e,f**) Control groups displayed normal tissue and cell morphology. (**g**) The preeclamptic placenta showed severe tissue damage and necrotic cells with loss of plasma membrane integrity and organelle breakdown, as well as extensive blebbing. Distorted mitochondria and dilated ER along with extensive cell debri was seen. (**h**) Human rA1M treatment protected tissue structure, membrane integrity and organelle morphology.

Histological analysis of placentas using hematoxylin & eosin (H&E) staining revealed an overall appearance of fuzzy and disrupted structures in the labyrinth zone of the placentas from the PE-buff group, with unclear boundaries between vessels and cells as well as areas of necrotic appearance (Fig. 3c and Supplementary Fig. S4). In this zone, there were also areas with both swollen cells and structures. Human rA1M treatment resulted in structures similar to the control placentas, with reduced swelling and no signs of necrosis (Fig. 3d). The control placentas displayed clear and well-defined structures over the whole labyrinth zone with no signs of necrosis or swollen structures (Fig. 3a,b). The structural changes observed in the preeclamptic placentas were more clearly visible by transmission electron microscopy (TEM) analysis, where morphological changes were demonstrated on the cellular level (Fig. 3g and Supplementary Fig. S5). The tissue showed signs of severe tissue disruption with cells displaying loss of plasma membrane integrity and organelle breakdown, as well as extensive amounts of apoptotic bodies. Swollen and distorted mitochondria and dilated ER were regularly observed. Empty extracellular space containing cell debris from dead cells indicated breakdown of structural components such as collagen fibers. Human rA1M treatment protected tissue structure, membrane integrity, and organelle morphology in preeclampsia (Fig. 3h). Also, no disrupted mitochondria or blebbing was seen, and apoptotic cells were rare. The placenta from both control groups displayed normal tissue morphology (Fig. 3e,f).

### Human rA1M alleviates the glomeruli damage observed in the kidneys

**Figure 4:**
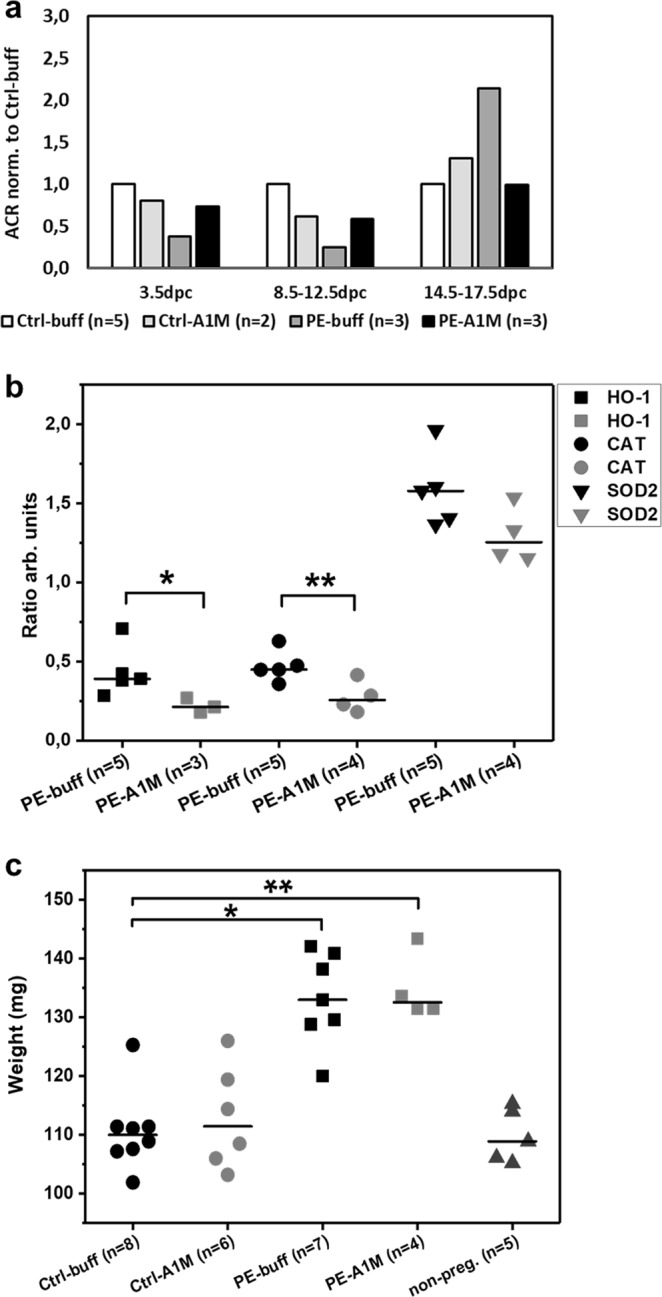
Human rA1M protects kidney function but no effect on cardiac hypertrophy. (**a**) Albumin/Creatinine ratio (ACR) analysis of urine from pregnant females showed an increase of ACR at late-gestation in the preeclamptic females, which was absent in the rA1M-treated group. Shown is the mean ACR normalised to Ctrl-buff values for each gestation period, and n = number of females analysed. (**b**) Gene expression levels for *HO-1*, *CAT* and *SOD2*, normalised to the *HPRT*-gene levels in kidney from PE-buff and PE-A1M females, demonstrating significant reduction of *HO-1* and *CAT* expression (*p = 0.04; **p = 0.04) after rA1M-treatment. The line represents the median and n = number of females analysed. (**c**) Preeclamptic females showed increased heart weight compared to Ctrl-buff at 17.5 dpc (*p = 0.002), which could not be alleviated by rA1M treatment (PE-A1M vs Ctrl-buff; **p = 0.008). Control groups showed similar heart weight as non-pregnant females. The line represents the median and n = number of females analysed.

**Figure 5:**
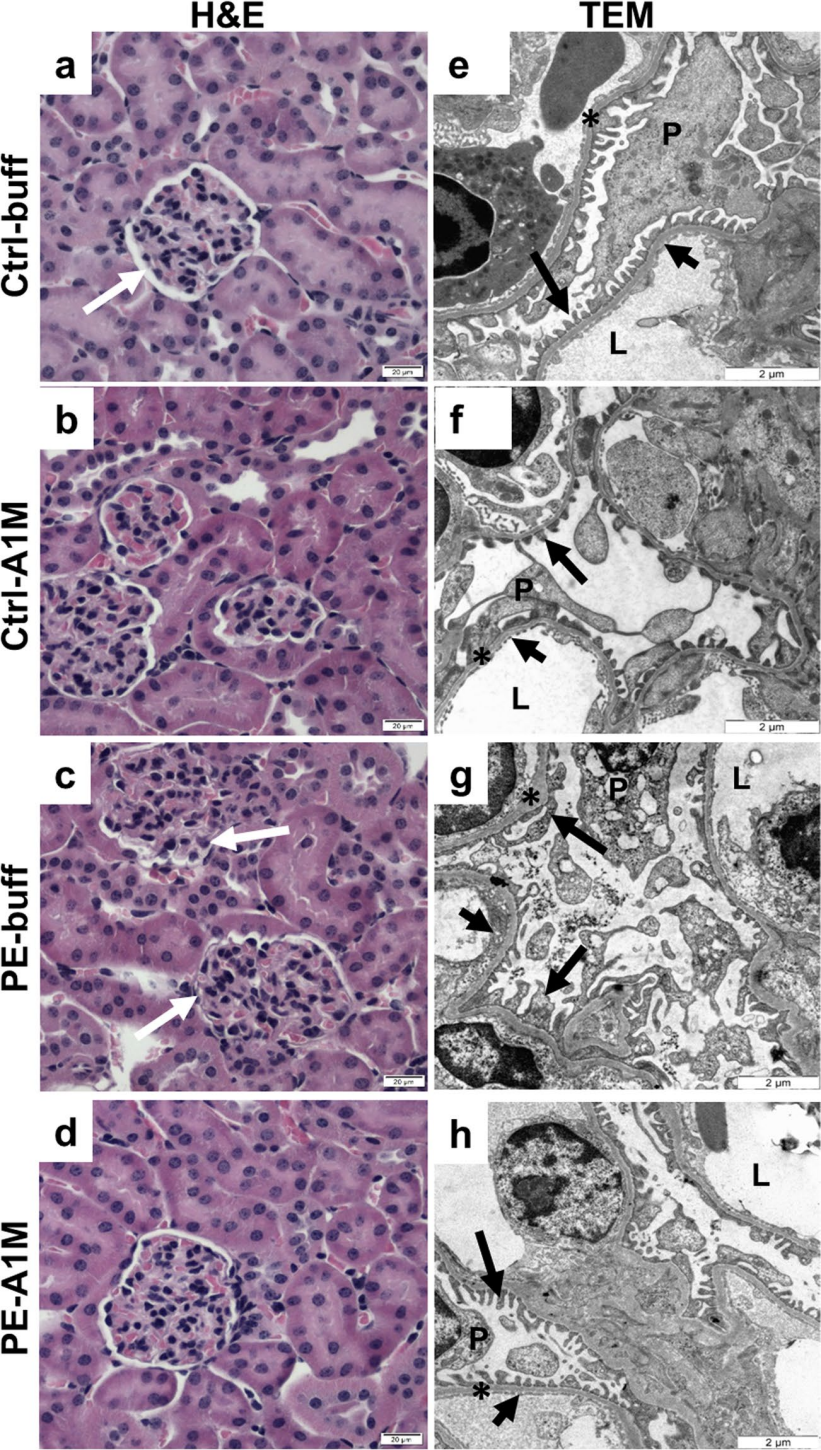
Histological and TEM analyses of kidney tissue structure. Morphological analysis of kidney biopsies at 17.5 dpc using H&E staining (**a**–**d**, scale bar = 20 µm) and TEM analysis (**e–h**, scale bar = 2 µm), showing representative images. (**a**–**b**) Control groups displayed normal tissue morphology. (**c**) Kidneys from preeclamptic females displayed glomerular tuft swelling resulting in reduced Bowman’s space. (**d**) Treatment with rA1M alleviated these glomerular changes to a level similar to the control groups. (**e,f**) Control groups displayed normal tissue and cell morphology. (**g**) Kidneys from preeclamptic females showed pathological changes including podocytes with intracellular vesicular bodies and disrupted mitochondria and ER, swollen and irregular glomerular basal membrane, effacement of podocyte foot processes and irregular and structurally aberrant endothelial fenestration. (**h**) Human rA1M-treatment protected the structure of the tissue, showing normal cell morphology and tissue organisation. White arrow = Bowman’s space, P = podocyte, L = lumen, *basal membrane, long black arrow = podocyte foot processes, short black arrow = endothelial fenestration.

The preeclamptic females developed a tendency to proteinuria at late gestation, which was not present in subjects receiving rA1M treatment (Fig. 4a). Histology analysis of kidneys from preeclamptic females revealed a tendency of glomerular tuft swelling (in 1–2 glomeruli per 10 examined), resulting in reduction of the Bowman’s space, indicative of glomerular endotheliosis as seen in human preeclampsia (Fig. 5c). Treatment with rA1M alleviated these glomerular changes (Fig. 5d) to the level of the control groups (Fig. 5a,b). The TEM analysis revealed tissue damage at the cellular level in the glomeruli of the preeclamptic females (Fig. 5g), present in all glomeruli analysed. The pathological changes included podocytes with swollen and disrupted mitochondria and ER, and with an abundance of intracellular vesicular bodies. There was effacement of podocyte foot processes seen as fused and irregular shapes. The glomerular basal membranes were swollen and irregular in thickness. The endothelial fenestration lining the basal membrane was irregular and structurally aberrant. The vascular lumen contained extracellular vesicles and lumen occlusion was evident. Treatment with rA1M protected the tissue, resulting in podocytes with normal cell morphology, podocyte foot processes that were not fused and regular in shape, as well as thinner basal membranes with smooth texture (Fig. 5h). The vascular lumen contained fewer vesicles and the fenestration showed normal frequency. The control groups displayed normal glomeruli morphology (Fig. 5e,f). The expression of genes involved in the protection against oxidative stress or apoptosis was analysed in kidney at day 17.5 dpc of preeclamptic females and rA1M-treated preeclamptic females (Fig. 4b). Human rA1M treatment resulted in significant reduction of both heme oxygenase-1 (*HO-1*) (p = 0.04) and catalase (*CAT*) (p = 0.04) levels, and a trend towards reduced superoxide dismutase 2 (*SOD2*) (p = 0.07) in kidneys from the PE-A1M group compared to the PE-buff group.

### Human rA1M alleviate cardiac tissue damage seen in the STOX1 preeclampsia model

**Figure 6:**
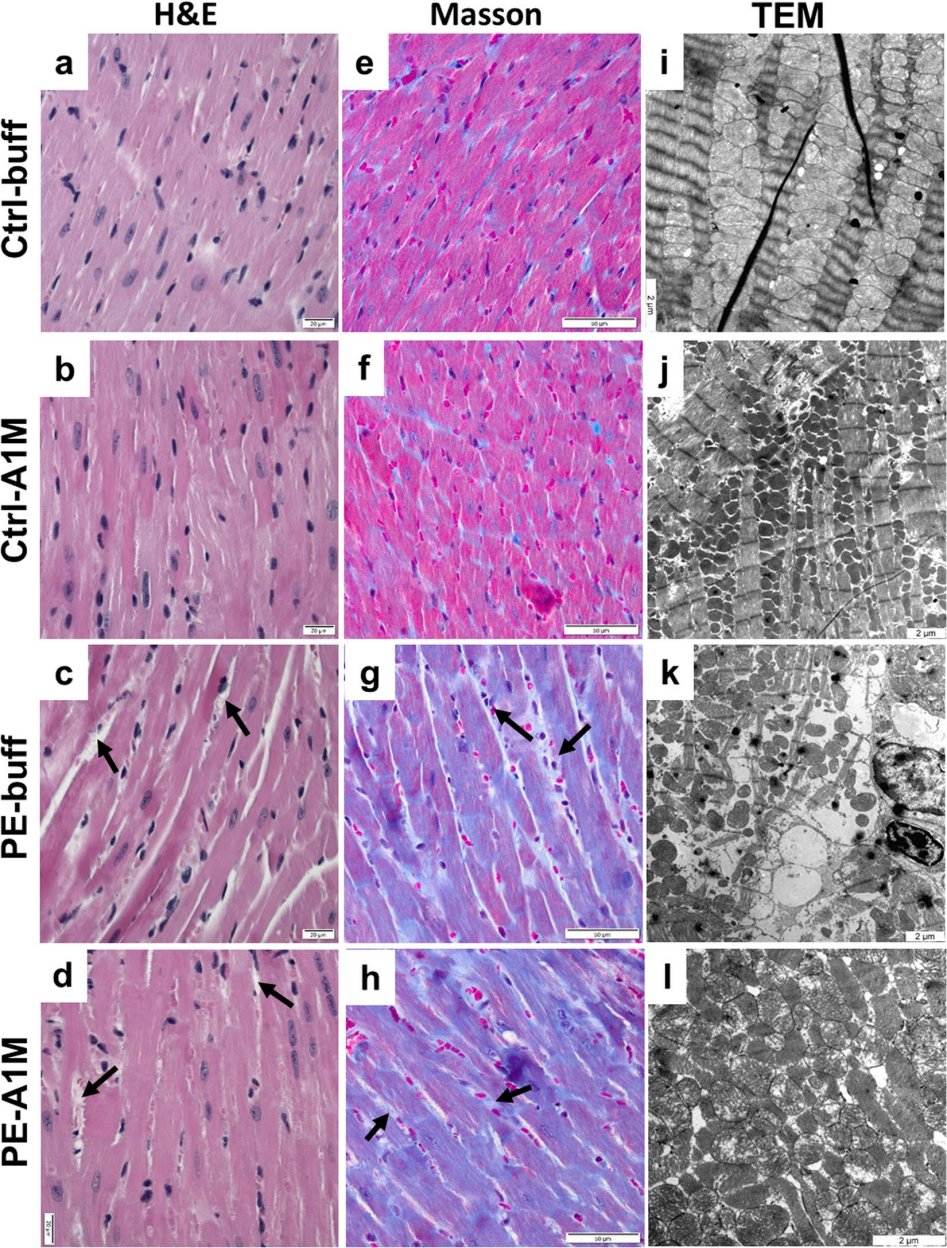
Histological and TEM analyses of heart tissue structure. Morphological analysis of heart biopsies at 17.5 dpc using H&E staining (**a**–**d**, scale bar = 20 µm), Masson trichrome staining (**e**–**h**, scale bar = 50 µm) and TEM analysis (**i**–**l**, scale bar = 2 µm), showing representative images. (**a**,**b**,**e,f**) Control groups displayed slender muscle fibers that were densely packed and stained pink. (**c)** Hearts from preeclamptic females displayed structural changes with increased extra-cellular space (arrows), cellular swelling and (**g**) an intense overall blue staining, indicating presence of collagen. (**d**,**h**) The same structural changes and intense Masson trichrome blue staining was seen in the rA1M-treated females. (**i,j**) The control groups displayed an organised structure between muscle fibers and healthy mitochondria. (**k**) The preeclamptic hearts had structural and cellular damages with swollen and erupted mitochondria, along with irregular organization of muscle fibers and mitochondria. Increased extra-cellular space could be observed in many places, indicating disrupted tissue. (**l**) Human rA1M treatment resulted in to some extent a more organized structure, with less mitochondrial damage.

The heart weight of the preeclamptic females was significantly increased at time of termination (17.5 dpc) compared to controls (p = 0.002) (Fig. 4c), which was not alleviated by rA1M treatment (PE-A1M vs Ctrl-buff; p = 0.008). At 17.5 dpc, both the control groups showed similar heart weight as non-pregnant females. Histology analysis using H&E staining of heart biopsies revealed that preeclampsia caused structural changes to the heart muscle, with increased extracellular space and swollen cells (Fig. 6c). This was also observed in the rA1M treated preeclampsia group, however with increased extracellular space to a lesser extent (Fig. 6d). Both control groups displayed slender muscle fibers that were densely packed together (Fig. 6a,b). Masson’s Trichrome staining revealed an intense overall blue staining for collagen being present throughout the whole biopsy from the preeclamptic heart (Fig. 6g and Supplementary Fig. S6), which was not alleviated by rA1M treatment (Fig. 6h). The TEM analysis confirmed that the preeclamptic heart showed structural and cellular damages, with the typical striated appearance missing to a large extent. Swollen and erupted mitochondria, along with irregular organization of muscle fibers and mitochondria were commonly seen, indicating disrupted tissue integrity (Fig. 6k and Supplementary Fig. S7). Human rA1M treatment could to some extent protect the cardiac macrostructure and cellular structures (Fig. 6l), with more organized striated muscle fiber structures and less mitochondrial damage. The control groups displayed an organized striated muscle fiber structure and densely packed mitochondria (Fig. 6i,j). Magnetic resonance imaging (MRI) analysis on a separate set of non-treated pregnant controls and preeclamptic females (not part of the treatment groups) revealed no significant difference in left or right ventricular end-diastolic or end-systolic volumes, left ventricular mass or ejection fraction between groups (Supplementary Table S1), although there was a trend towards increased left ventricular mass in mice with preeclampsia. Left ventricular cardiac output was increased in the preeclampsia group (p = 0.029) combined with a trend towards an increased heart rate in these females.

### Blood analysis

Human rA1M treatment had no alleviating effect on the plasma levels of sFlt1, or sEng in the preeclamptic females (Supplementary Fig. S8).

## Discussion

In the present study, we analysed the structural, ultrastructural, and functional alterations induced by preeclampsia on the maternal organs and the placenta, and the therapeutic effect of rA1M on these changes.

Our results demonstrate the potential therapeutic capacity of rA1M to alleviate typical manifestations of preeclampsia, such as elevated BP, proteinuria, and organ damage. Similar protective effects have previously been demonstrated in other animal models of preeclampsia^30,31^, with reduced proteinuria, and reverted kidney and placenta damage. However, in this study we use a preeclampsia animal model to demonstrate a significant effect by rA1M on lowering an elevated BP, as well as proteinuria. The strongest therapeutic effect on elevated BP was seen mid-gestation, but also to some extent at late-gestation. We used the same dose of A1M throughout the experiments, and a higher dose might have been needed at late-gestation to have the same strong effect. Endogenous A1M has been shown to be up-regulated in women with preeclampsia^32^, suggesting that it plays a role in the endogenous defense against increased oxidative and nitrative stress. By supplementing with rA1M, the suggested therapy mimics nature’s own way of responding to the problem. Today, aspirin is used as a prophylactic treatment in high-risk pregnancies to prevent preeclampsia but is only effective when it is administered in early pregnancy before the symptoms appear, as a prophylaxis^20^. Clearly, a therapeutic molecule, able to reverse the symptoms once they appear is missing. Currently, to cure preeclampsia, delivery remains the only option. New research approaches, such as apheresis to extract excess sFlt1 from the maternal blood have been proposed^33^. However, such a method is expensive and likely not suitable for a broad clinical implementation, particularly not in low- and middle-income countries, where preeclampsia is a large problem. Human rA1M treatment could be a promising alternative, since it is an endogenous protein, naturally produced by the liver that could be supplemented to patients without fearing severe side effects.

The protective effects of rA1M treatment regarding both elevated BP and oxidative and nitrative stress supports its role as an endogenous radical-scavenging and tissue repair protein^27^. It has been shown that A1M has a protective role against oxidative stress and binds to mitochondria to help maintain morphological structure and ATP production^34,35^. The reduction in kidney expression levels of *HO-1*, *CAT* and *SOD2* in the rA1M-treated females supports this protective role, since these are genes involved in responses towards oxidative stress or apoptosis. HO-1 is a stress response protein and is upregulated in response to its substrate haem, but also to oxidative stress-inducing factors such as ROS^36^. CAT is involved in the protection of cells from oxidative damage by hydrogen peroxidase^37^, while SOD2 plays an anti-apoptotic role against oxidative stress by clearing mitochondrial ROS and protecting against cell death^38^. In this study, A1M may stabilize the hyperactive mitochondria, reduce their increased free radical production, and reduce activation of NO production pathways, which in turn would lead to reduced levels of peroxynitrite and nitrotyrosine. Experiments have demonstrated a number of nitrated proteins being present in the normal placenta^39^, where it is thought to occur as a posttranslational modification of proteins^24^. For preeclampsia, heightened levels of oxidative and nitrative stress has been reported^7,10^, elevated levels of peroxynitrite^25^, and an abundance of nitrotyrosine residues are present in the placenta^10,40,41^. Elevated levels of peroxynitrite can result in significantly altered function of proteins and mitochondria, as well as inflicting damage to nucleic acids. Nitrated proteins have also been reported to be able to elicit immune responses and to be involved in autoimmune diseases^42,43^.

In this study, we could not detect foetal intra uterine growth restriction at day 17.5 dpc, as has previously been reported for days 17.5 and E18.5 dpc, but not for 16.5^44^. This observation could be due to breeding conditions, leading to difference in growth kinetics. However, in the present study, we could detect a significant decrease of placental weight as a result of preeclampsia. The placental weight was normalized by rA1M treatment, probably by protection of the placenta against oxidative/nitrative stress, and thereby reducing their damaging effects on the tissue integrity. This would result in a more normal placental function. Since before, A1M has been shown to both protect and repair tissue from oxidative lesions^27^. Furthermore, we used cardiac MRI to evaluate the effect of preeclampsia on functional properties of the left and right ventricle, but found no decrease in left ventricular ejection fraction in the preeclamptic females as previously reported^44^. An increase in left ventricular cardiac output was shown, which is in line with prior clinical studies describing a subgroup of preeclamptic women with increases in cardiac output in preeclampsia^45^. Further, a slight increase in left ventricular mass was found in preeclamptic females, which consisted of a general hypertrophy including the papillary muscles and trabeculation. To have a more definitive answer regarding the effect of preeclampsia on myocardial hypertrophy and cardiac function, rigorous adjustments taking into account individual cardiac and body weights, and other known confounding factors, as well as an increase in the number of animals analysed would be required. Nevertheless, we find the observation consistent with increase of heart mass and anomalies of its structure.

Human rA1M protected tissue integrity in the kidneys, resulting in normal glomeruli morphology and reduced proteinuria, in line with previous *in vivo* studies^30,31^. The heart of the preeclamptic females showed hypertrophy similar to what has been reported for preeclamptic women^18^, and consistent with our previous observations in the STOX1 model^22^. This was seen as altered tissue morphology, with swollen mitochondria and disrupted muscle fibers with collagen, demonstrating that oxidative stress in the placenta can cause alterations in the heart. Human rA1M protected the mitochondria and reduced the extent of disrupted cardiac tissue. However, the weight of the heart was not reduced by rA1M treatment, probably due to the persisting collagen. This is in line with reports for a subgroup of preeclamptic women that develop persistent alterations in cardiac function as a consequence of the disease^46^. The reason for this is poorly understood. As previously shown in a mouse model, overexpression of sFlt-1 has no long-term effect on BP and vascular function in the postpartum mothers^47^. Thus, collagen deposits and mitochondrial damage may provide a better explanation for long-term cardiac effects of preeclampsia. Overall, compared to kidneys and placentas, the heart was protected to a lesser extent by rA1M treatment. The exact mechanisms behind the development of these heart alterations needs to be further studied, in combination with rA1M treatment at earlier time points. The considerable protection of the kidney is of importance, as this was both structural and functional. It is established that preeclampsia induces long-term deleterious cardiovascular and renal consequences for the mothers^48–50^, and protecting the organs already during pregnancy is likely crucial. We have shown in a rabbit preeclampsia model and in women with preeclampsia that urinary shedding of extracellular vesicles from podocytes is increased and correlates with the renal injury and proteinuria^51^. Furthermore, human rA1M-treatment of the pregnant rabbits had a protective role and reduced the levels of both Annexin-V^+^ and Podocin^+^ vesicles in urine (unpublished data). We therefore hypothesis that rA1M could be efficient for preserving normal organ function in general, and kidney function specifically, improving the long-term function.

This study confirms that rA1M has a protective role against both organ damage and secondary effects as elevated BP in preeclampsia. Recombinant A1M thus has potential as a therapeutic drug in preeclampsia and likely also in other pathological conditions associated with oxidative stress.

## Methods

### Ethics committee approval

This study used the STOX1 transgenic mouse model on FVB/N strain background^21^, and was approved by the local ethics committee for animal studies at Lund University, Lund, Sweden (permit no: M29-14) and Institut National de Recherche Agronomique (INRA), Jouy-en-Josas, France (permit no: 12/035 (06.29.2012)). Mice were kept at the Bio Medical Center (BMC) animal facility, Lund University, Sweden or at the animal facility of INRA, Jouy en Josas (outside Paris), France in a controlled environment (light/dark cycle, temperature, free access to food and water). Animal experiments were carried out in strict accordance with the recommendations in the guidelines of the Code for Methods and Welfare Considerations in Behavioural Research with Animals (Directive 86/609EC). All efforts were made to minimize suffering.

### Human recombinant A1M

Human rA1M was donated by A1M Pharma AB (Lund, Sweden). The full polypeptide of human plasma A1M preceded by an N-terminal His8-tag was prepared as previously described^52^, and tested for activity^53^. The rA1M solution was dissolved in 10 mM Tris-HCl pH 8.0, 0.125 M NaCl at a concentration of 2.25 mg/ml (0.08 EU/mg) and sterile filtered. The solution was kept at −80 °C until use. Mice were injected i.p. with 0.27 mg rA1M. The control mice were injected with an equal volume of buffer (10 mM Tris-HCl pH 8.0, 0.125 M NaCl). Human rA1M was detected in mouse plasma after the i.p. injections by using an in-house human A1M-specific radioimmunoassay (RIA) described previously^32^.

### Experimental set-up

Figure 1 illustrates the experimental design. The four experimental groups were two control groups (wt females mated to wt males and injected with buffer during pregnancy (Ctrl-buff) and wt females mated to wt males and injected with rA1M during pregnancy (Ctrl -A1M)), and two preeclampsia groups (wt females mated to transgenic males and injected with buffer during pregnancy (PE-buff) and wt females mated to transgenic males and injected with rA1M during pregnancy (PE-A1M)). The females used were 10–20 weeks old. The experiment lasted from time of mating until termination at gestation day 17.5 dpc. The females were given six i.p. injections of either buffer or rA1M every second day starting at 6.5 dpc. BP (systolic and diastolic) was measured every second day, starting before mating to get a baseline and throughout the experiment (for ~35 consecutive days per mouse), by a non-invasive tail-cuff device (CODA8 with four channels, EMKA Technologies). Non-anesthetized mice, previously trained for 1 week to the manipulation, were placed in animal restrainers of appropriate size and placed on a warming platform. The system uses volume pressure recording sensors and an occlusion tail-cuff to repeatedly determine changes in the tail volume, corresponding to systolic and diastolic pressure, with at least 3 satisfactory measurements per day. Systolic and diastolic BP always displayed similar curve profiles in all groups analysed, therefore only systolic BP is shown. Urine was collected during the experiment non-invasively by putting female mice on a cold metal surface to induce urination, and stored at −80 °C until use. However, urine samples were difficult to collect from all females at every time point, and therefore results were pooled within each group for each gestational period (early, mid and late). Whole blood was collected from the Saphena vein at early- and mid-gestation, and from the Cava vein at time of termination in Li-Heparin tubes. Plasma was separated from whole blood and then stored at −80 °C until use. Females were sacrificed at day 17.5 dpc and organs collected. The organs were dissected and biopsies were fresh-frozen on dry ice, paraffin-embedded, or fixed for TEM. Pups were euthanized, counted, and weighed. The placentas and maternal hearts were weighed. Differences in number of animals per group are related to different experimental locations; Paris, France and Lund, Sweden (Supplementary Table S2). The following data analysis were performed on mouse experiments executed in Paris: BP measurements, Nitrotyrosine analysis, hA1M-RIA analysis, qPCR analysis, and plasma sEng/sFlt1 analysis. The following data analysis were performed on mouse experiments executed in Lund: Hypoxyprobe analysis, HE/Masson/TEM microscopy analysis, ACR analysis and MRI analysis. Heart weight was included from both locations, explaining the higher n-numbers.

### Gene expression analysis in kidney

Total RNA was purified from frozen kidney biopsies using TRIzol (Life Technologies) followed by an E.Z.N.A. Total RNA kit (Omega Bio-Tek, VWR). Reverse transcription with random hexamers was done using TaqMan Reverse Transcription kit (Applied Biosystems, Roche). Real-time PCR was performed for *HO-1*, *CAT*, *SOD2*, with hypoxanthine phosphoribosyltransferase (*HPRT)* as endogenous control, using TaqMan Gene Expression Assays specific for mouse (*HO-1* – Mm00516005_m1, *CAT* – Mm00437992_m1, *SOD2* – Mm01313000_m1, *HPRT* – Mm01545399_m1, Life Technologies). The analyses were done using the relative standard curve method, where a 4-fold dilution series of a complementary DNA (cDNA) from mouse placenta was used as an arbitrary standard. This was used to give an arbitrary unit for each sample as defined by a standard curve for each gene tested. The arbitrary unit for the gene of interest was normalized against the value for *HPRT* to give an expression ratio that could be compared between samples. All samples were run as duplicates.

### Blood and urine analysis

The plasma was analysed for sFlt1 using a Quantikine Mouse VEGF R1/Flt-1 Immunoassay (R&D Systems), and for sEng using a Quantikine Mouse Endoglin/CD105 Immunoassay (R&D Systems). The level of proteinuria in urine was measured as the albumin/creatinine ratio (ACR) at early, mid and late gestation, by combining a murine urinary albumin ELISA kit (Albuwell M, Exocell) and a creatinine chemical assay (the Creatinine Companion, Exocell). For all analysis, samples were run in duplicates.

### Hypoxyprobe

For detection of tissue hypoxia, we used the hypoxia marker pimonidazole hydrochloride, also known as Hypoxyprobe-1 (Hypoxyprobe Inc.). Pimonidazole is reductively activated in hypoxic cells and form stable adducts with thiol groups in proteins in both normal and malignant tissue. A monoclonal antibody binds specifically to formed adducts, allowing their detection by immunoperoxidase analysis of formalin-fixed paraffin-embedded sections. Two hours before termination on day 17.5 dpc, Hypoxyprobe-1 (at 116 mg/ml in 0.9% saline solution) was injected i.p. in pregnant females at 60 mg/kg body weight. Placentas were cut through the mid-section, formalin-fixed and paraffin-embedded for immunohistochemistry using peroxidase detection on sections according to manufacturer’s protocol. On average, four placentas per female were analysed and stained sections were scanned as 20x magnification high resolution images using a Hamamatsu Nanozoomer S60 scanner (Hamamatsu Photonics). To quantify the peroxidase staining in the placenta, an algorithm was developed using the ImageJ software platform, where positive peroxidase stained area was calculated as percent of the whole area of the specimen (any holes excluded).

### Protein nitration assay

To detect nitrotyrosine recidues on nitrated proteins in placenta, we used the OxiSelect Nitrotyrosine ELISA kit (Cell Biolabs Inc.) according to manufacturer’s protocol. Protein extractions from placenta were prepared by adding homogenizing buffer (50 mM Tris-HCl pH 8.0, 2 mM EDTA, 0.5% NP-40, one Complete mini protease inhibitor cocktail tablet/10 ml buffer (Roche)) to biopsies and sonicated for 10 s. The tissue was further shredded by pipetting and thereafter centrifuged at 10000 × g for 30 min. The supernatant was collected and protein concentration measured by Pierce BCA Protein assay kit (Thermo scientific).

### Histology

For histology analysis of tissue morphology, we used H&E staining according to standard protocols, or Masson’s Trichrome staining (a three-color staining, including Aniline Blue that is specific for collagen) according to manufacturer’s protocol (Reactifs RAL) on formalin-fixed paraffin-embedded biopsies. Biopsies were sectioned at four µm thickness using standard protocols and morphology was evaluated by light microscopy using an Olympus BX60 microscope with cellSens Entry micro imaging software. Microscopy was performed on sections from four placentas, one kidney and the heart per female and with two or three females per experimental group.

### Transmission electron microscopy

Biopsies from placenta, kidney, and heart (3 × 3 × 3 mm) were fixed for two hours at room temperature in fixative (1.5% paraformaldehyde and 1.5% glutaraldehyde in 0.1 M Sörensen buffer pH 7.2), washed and stored overnight at 4 °C in Sörensen buffer. The fixed samples were prepared for ultrathin sectioning and subjected to TEM as reviewed in^54^. Microscopy was performed on duplicate sections from one biopsy per organ and female, with two-three females per experimental group. For kidney sections, 3–5 glomeruli were present per section.

### Magnetic resonance imaging analysis

MRI experiments were performed on pregnant control and preeclamptic females (not included in the treatment groups) at gestational day 17.5 dpc using a 9.4 T horizontal bore MR scanner (Bruker, Germany) with the sedated pregnant mouse positioned on a warming pad to maintain constant body temperature at 36–37 °C. A prospectively electrocardiogram and respiration-triggered fast low angle shot (FLASH) sequence was used to acquire cine MR images in a short-axis stack covering the ventricles and two- and four-chamber views. To ensure full coverage of the cardiac cycle, 24 frames with a temporal resolution of 6 ms were acquired. Imaging parameters were field of view 25 × 25 mm, matrix 192 × 192, slice thickness 1 mm, no gap, echo time 2.1 ms, repetition time 6 ms, flip angle 15°, and number of averages 1. Manual segmentation of left and right ventricular endocardial borders and left ventricular epicardial borders at end-diastole and end-systole were performed in Segment v2.0 (Medviso AB, Sweden)^55^ for end-diastolic and end-systolic volumes, ejection fraction, cardiac output and left ventricular mass. For left ventricular mass the papillary muscles and trabeculation were included as myocardium to quantify potential hypertrophy related to preeclampsia.

### Statistical analysis

All data were analysed by Origin 8 software (Microcal Northampton, USA). Data are presented as mean ± SEM or as median (range), where appropriate. Differences between groups were evaluated using the 2-sample Student’s *t*-test with Welch corrections (Fig. 2A) and Mann-Whitney U-test (all other figures and tables). P-values of p = 0.05 were considered statistically significant. No statistical test was performed for ACR-values in urine (Fig. 4) due to pooled and normalised results.

## Supplementary information

Supplementary Information

## Data Availability

Materials, data and associated protocols are available upon request.

## References

[1] Myatt L, Roberts JM (2015). Preeclampsia: Syndrome or Disease?. Current hypertension reports.

[2] Sibai B, Dekker G, Kupferminc M (2005). Pre-eclampsia. Lancet.

[3] Steegers EA, von Dadelszen P, Duvekot JJ, Pijnenborg R (2010). Pre-eclampsia. Lancet.

[4] Burton GJ, Jauniaux E (2004). Placental oxidative stress: from miscarriage to preeclampsia. Journal of the Society for Gynecologic Investigation.

[5] Borzychowski AM, Sargent IL, Redman CW (2006). Inflammation and pre-eclampsia. Seminars in fetal & neonatal medicine.

[6] Redman CW, Sacks GP, Sargent IL (1999). Preeclampsia: an excessive maternal inflammatory response to pregnancy. American journal of obstetrics and gynecology.

[7] Roberts JM, Hubel CA (1999). Is oxidative stress the link in the two-stage model of pre-eclampsia?. Lancet.

[8] Wang Y, Walsh SW (2001). Increased superoxide generation is associated with decreased superoxide dismutase activity and mRNA expression in placental trophoblast cells in pre-eclampsia. Placenta.

[9] Mazzanti L (2012). Nitric oxide and peroxynitrite platelet levels in gestational hypertension and preeclampsia. Platelets.

[10] Myatt L (1996). Nitrotyrosine residues in placenta. Evidence of peroxynitrite formation and action. Hypertension.

[11] Gorlach A, Klappa P, Kietzmann T (2006). The endoplasmic reticulum: folding, calcium homeostasis, signaling, and redox control. Antioxidants & redox signaling.

[12] Malhotra JD (2008). Antioxidants reduce endoplasmic reticulum stress and improve protein secretion. Proceedings of the National Academy of Sciences of the United States of America.

[13] Morris JM (1998). Circulating markers of oxidative stress are raised in normal pregnancy and pre-eclampsia. British journal of obstetrics and gynaecology.

[14] Toescu V, Nuttall SL, Martin U, Kendall MJ, Dunne F (2002). Oxidative stress and normal pregnancy. Clinical endocrinology.

[15] Wisdom SJ, Wilson R, McKillop JH, Walker JJ (1991). Antioxidant systems in normal pregnancy and in pregnancy-induced hypertension. American journal of obstetrics and gynecology.

[16] Goulopoulou S, Davidge ST (2015). Molecular mechanisms of maternal vascular dysfunction in preeclampsia. Trends in molecular medicine.

[17] Bello N, Rendon ISH, Arany Z (2013). The relationship between pre-eclampsia and peripartum cardiomyopathy: a systematic review and meta-analysis. Journal of the American College of Cardiology.

[18] Melchiorre K, Sutherland GR, Liberati M, Thilaganathan B (2011). Preeclampsia is associated with persistent postpartum cardiovascular impairment. Hypertension.

[19] Bellamy L, Casas JP, Hingorani AD, Williams DJ (2007). Pre-eclampsia and risk of cardiovascular disease and cancer in later life: systematic review and meta-analysis. Bmj.

[20] Rolnik DL (2017). Aspirin versus Placebo in Pregnancies at High Risk for Preterm Preeclampsia. The New England journal of medicine.

[21] Doridot Ludivine, Passet Bruno, Méhats Céline, Rigourd Virginie, Barbaux Sandrine, Ducat Aurélien, Mondon Françoise, Vilotte Marthe, Castille Johann, Breuiller-Fouché Michelle, Daniel Nathalie, le Provost Fabienne, Bauchet Anne-Laure, Baudrie Véronique, Hertig Alexandre, Buffat Christophe, Simeoni Umberto, Germain Guy, Vilotte Jean-Luc, Vaiman Daniel (2013). Preeclampsia-Like Symptoms Induced in Mice by Fetoplacental Expression of STOX1 Are Reversed by Aspirin Treatment. Hypertension.

[22] Ducat A (2016). Endothelial cell dysfunction and cardiac hypertrophy in the STOX1 model of preeclampsia. Scientific reports.

[23] Doridot L (2014). Nitroso-redox balance and mitochondrial homeostasis are regulated by STOX1, a pre-eclampsia-associated gene. Antioxidants & redox signaling.

[24] Pacher P, Beckman JS, Liaudet L (2007). Nitric oxide and peroxynitrite in health and disease. Physiological reviews.

[25] Roggensack AM, Zhang Y, Davidge ST (1999). Evidence for peroxynitrite formation in the vasculature of women with preeclampsia. Hypertension.

[26] Webster RP, Brockman D, Myatt L (2006). Nitration of p38 MAPK in the placenta: association of nitration with reduced catalytic activity of p38 MAPK in pre-eclampsia. Molecular human reproduction.

[27] Akerstrom B, Gram M (2014). A1M, an extravascular tissue cleaning and housekeeping protein. Free radical biology & medicine.

[28] Yu H (1983). Alpha-1-microglobulin: an indicator protein for renal tubular function. Journal of clinical pathology.

[29] May K (2011). Perfusion of human placenta with hemoglobin introduces preeclampsia-like injuries that are prevented by alpha1-microglobulin. Placenta.

[30] Naav A (2015). A1M Ameliorates Preeclampsia-Like Symptoms in Placenta and Kidney Induced by Cell-Free Fetal Hemoglobin in Rabbit. PloS one.

[31] Wester-Rosenlof L (2014). A1M/alpha1-microglobulin protects from heme-induced placental and renal damage in a pregnant sheep model of preeclampsia. PloS one.

[32] Olsson MG (2010). Increased levels of cell-free hemoglobin, oxidation markers, and the antioxidative heme scavenger alpha(1)-microglobulin in preeclampsia. Free radical biology & medicine.

[33] Thadhani Ravi, Hagmann Henning, Schaarschmidt Wiebke, Roth Bernhard, Cingoez Tuelay, Karumanchi S. Ananth, Wenger Julia, Lucchesi Kathryn J., Tamez Hector, Lindner Tom, Fridman Alexander, Thome Ulrich, Kribs Angela, Danner Marco, Hamacher Stefanie, Mallmann Peter, Stepan Holger, Benzing Thomas (2015). Removal of Soluble Fms-Like Tyrosine Kinase-1 by Dextran Sulfate Apheresis in Preeclampsia. Journal of the American Society of Nephrology.

[34] Akerstrom B (2017). The Role of Mitochondria, Oxidative Stress, and the Radical-binding Protein A1M in Cultured Porcine Retina. Curr Eye Res.

[35] Olsson MG (2013). The radical-binding lipocalin A1M binds to a Complex I subunit and protects mitochondrial structure and function. Antioxidants & redox signaling.

[36] Elbirt KK, Bonkovsky HL (1999). Heme oxygenase: recent advances in understanding its regulation and role. Proc Assoc Am Physicians.

[37] Chelikani P, Fita I, Loewen PC (2004). Diversity of structures and properties among catalases. Cellular and molecular life sciences: CMLS.

[38] Pias EK (2003). Differential effects of superoxide dismutase isoform expression on hydroperoxide-induced apoptosis in PC-12 cells. The Journal of biological chemistry.

[39] Webster RP, Roberts VH, Myatt L (2008). Protein nitration in placenta - functional significance. Placenta.

[40] Bosco C (2012). Oxidative damage to pre-eclamptic placenta: immunohistochemical expression of VEGF, nitrotyrosine residues and von Willebrand factor. The journal of maternal-fetal & neonatal medicine: the official journal of the European Association of Perinatal Medicine, the Federation of Asia and Oceania Perinatal Societies, the International Society of Perinatal Obstet.

[41] Myatt L (2010). Review: Reactive oxygen and nitrogen species and functional adaptation of the placenta. Placenta.

[42] Hsu HC (2006). Production of a novel class of polyreactive pathogenic autoantibodies in BXD2 mice causes glomerulonephritis and arthritis. Arthritis Rheum.

[43] Khan F, Siddiqui AA (2006). Prevalence of anti-3-nitrotyrosine antibodies in the joint synovial fluid of patients with rheumatoid arthritis, osteoarthritis and systemic lupus erythematosus. Clinica chimica acta; international journal of clinical chemistry.

[44] Collinot H (2018). Preeclampsia induced by STOX1 overexpression in mice induces intrauterine growth restriction, abnormal ultrasonography and BOLD MRI signatures. Journal of hypertension.

[45] Carlsson C (1984). Cardiovascular changes in pre-eclampsia. Acta Obstet Gynecol Scand Suppl.

[46] Ghossein-Doha C (2013). Hypertension after preeclampsia is preceded by changes in cardiac structure and function. Hypertension.

[47] Bytautiene E (2010). Long-term maternal cardiovascular function in a mouse model of sFlt-1-induced preeclampsia. American journal of physiology. Heart and circulatory physiology.

[48] Irgens HU, Reisaeter L, Irgens LM, Lie RT (2001). Long term mortality of mothers and fathers after pre-eclampsia: population based cohort study. Bmj.

[49] Jarvie JL, Metz TD, Davis MB, Ehrig JC, Kao DP (2018). Short-term risk of cardiovascular readmission following a hypertensive disorder of pregnancy. Heart.

[50] Vikse BE, Irgens LM, Leivestad T, Skjaerven R, Iversen BM (2008). Preeclampsia and the risk of end-stage renal disease. The New England journal of medicine.

[51] Gilani SI (2017). Urinary Extracellular Vesicles of Podocyte Origin and Renal Injury in Preeclampsia. Journal of the American Society of Nephrology: JASN.

[52] Kwasek A (2007). Production of recombinant human alpha1-microglobulin and mutant forms involved in chromophore formation. Protein expression and purification.

[53] Akerstrom B, Maghzal GJ, Winterbourn CC, Kettle AJ (2007). The lipocalin alpha1-microglobulin has radical scavenging activity. The Journal of biological chemistry.

[54] Carlemalm E (1990). Lowicryl resins in microbiology. Journal of structural biology.

[55] Heiberg E (2010). Design and validation of Segment–freely available software for cardiovascular image analysis. BMC Med Imaging.

