# Supplementary material for: Alpha-1 microglobulin as a potential therapeutic candidate for treatment of hypertension and oxidative stress in the STOX1 preeclampsia mouse model
Source: Sci Rep. 2019 Jun 12;9:8561. doi: 10.1038/s41598-019-44639-9 (PMC6561956; doi:10.1038/s41598-019-44639-9)
Supplement: Supplementary file 1 — Supplementary Information [file 41598_2019_44639_MOESM1_ESM.pdf]

Alpha-1 microglobulin as a potential therapeutic candidate for treatment of hypertension and oxidative stress in the STOX1 preeclampsia mouse model

Lena Erlandsson[1*], Aurélien Ducat[2], Johann Castille[3], Isac Zia[1], Grigorios Kalapotharakos[1], Erik Hedström[4, 5], Jean-Luc Vilotte[3], Daniel Vaiman[2] and Stefan R. Hansson[1]

[1]*Obstetrics and Gynecology, Department of Clinical Sciences Lund, Lund University, Lund, Sweden.*

[2]*INSERM U1016, CNRS UMR8104, Faculté de Médecine, Institut Cochin, Paris, France.*

[3]*INRA-AgroParisTech, UMR1313 Génétique Animale et Biologie Intégrative, Institut National de la Recherche Agronomique, Jouy-en-Josas, France.*

[4]*Clinical Physiology, Department of Clinical Sciences Lund, Lund University, Lund, Sweden.*

[5]*Diagnostic Radiology, Department of Clinical Sciences Lund, Lund University, Lund, Sweden.*

* Corresponding author

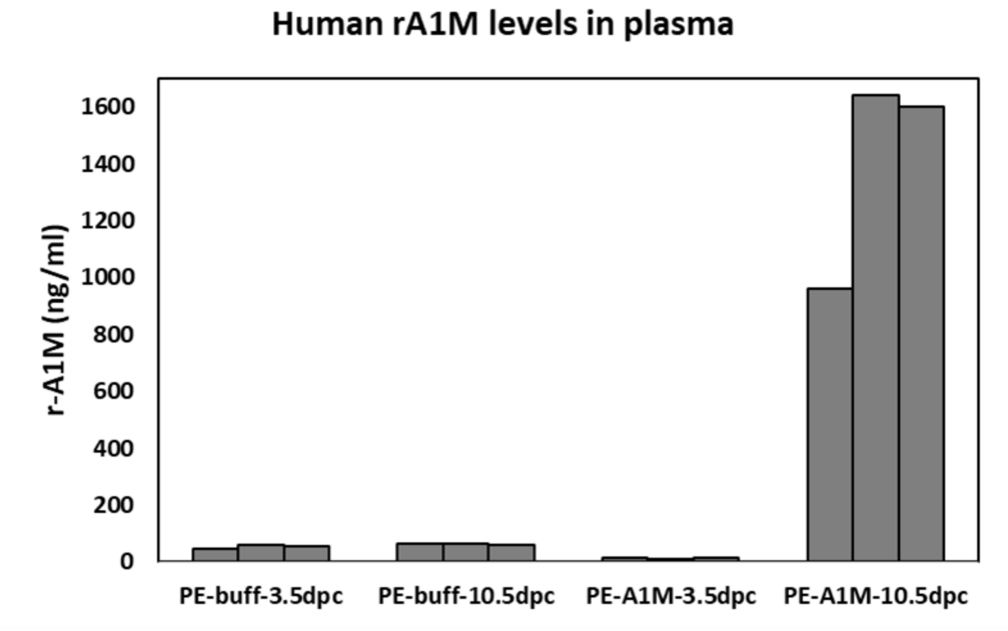

Figure S1. Detection of human rA1M in plasma from i.p.-injected pregnant females. A human A1M-specific RIA was used to demonstrate the presence of human rA1M in plasma from i.p.-injected pregnant females at gestational age 10·5 dpc (PE-A1M). Human rA1M was obviously not present prior to injections (3·5 dpc) or in the PE-buff group. Shown is results for 3 females per group.

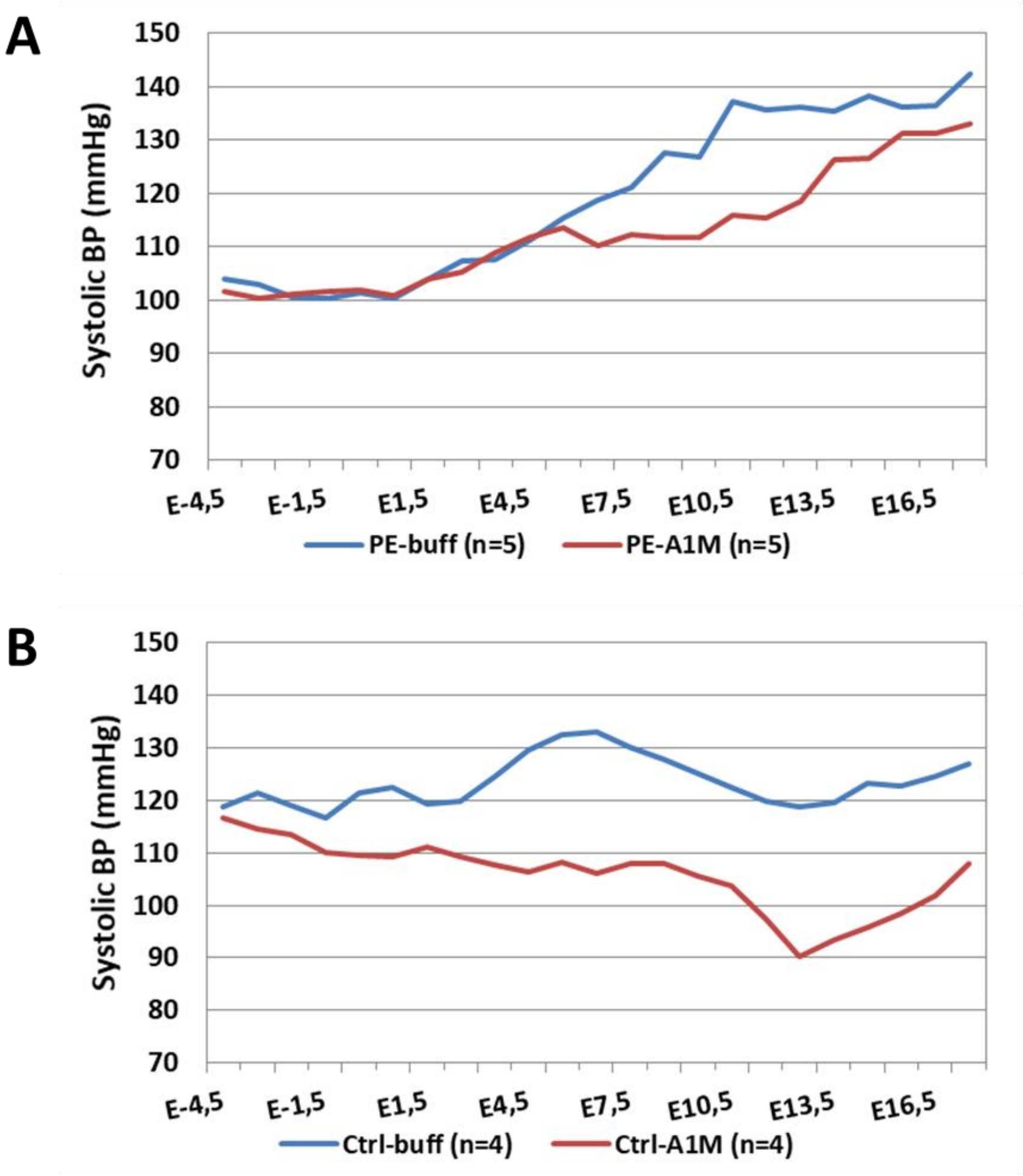

Figure S2. Systolic BP (mmHg) throughout gestation.

Absolut values for systolic BP shown in 5-days sliding windows for (A) PE-buff and PE-A1M groups and for (B) Ctrl-buff and Ctrl-A1M groups. n=number of females analysed.

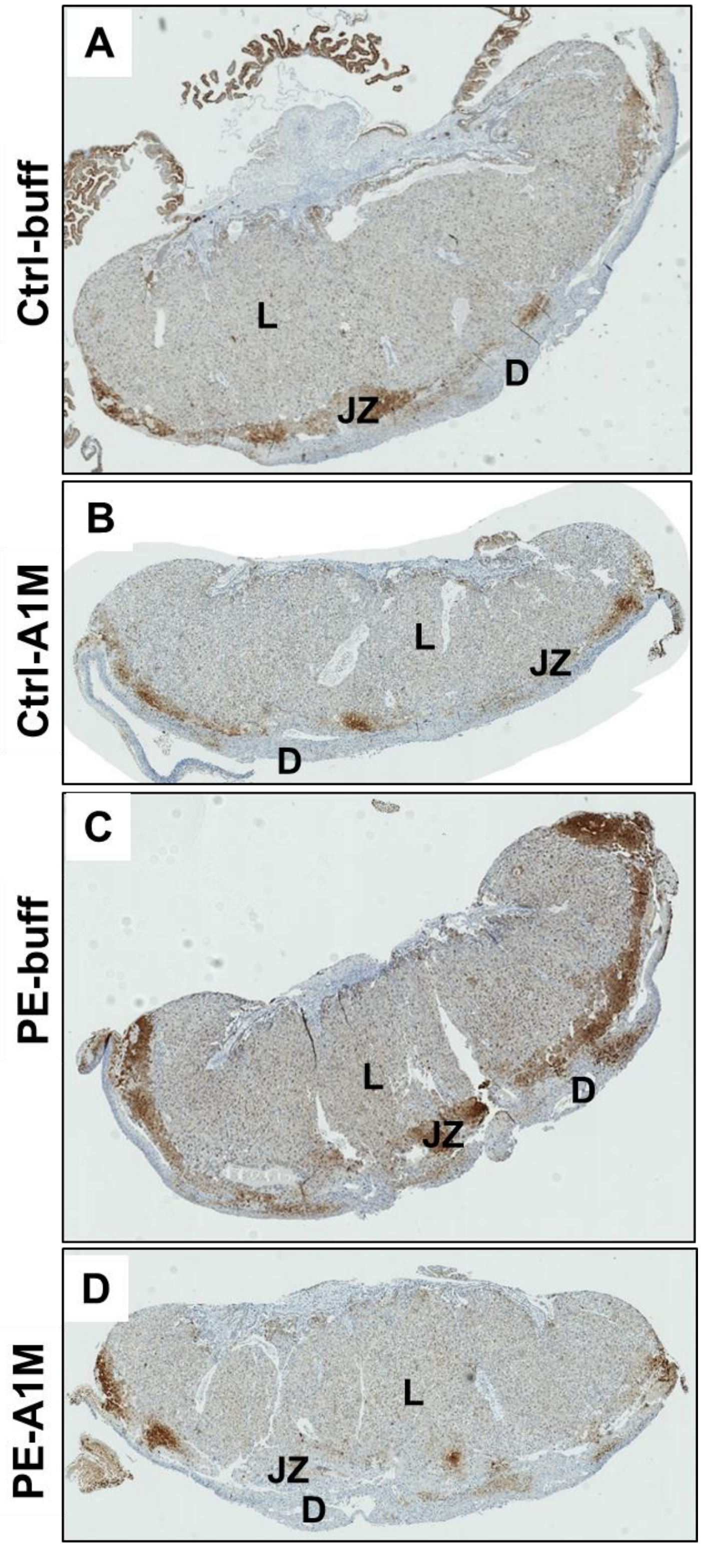

Figure S3. Hypoxyprobe immunohistochemistry in mouse placenta.

Representative images showing Hypoxyprobe immunohistochemistry in mouse placenta. A: Ctrl-buff. B: Ctrl-A1M. C: PE-buff. D: PE-A1M. Strong staining was found in the junctional zone of the placenta. The percentage area of positive staining was determined using ImageJ software. JZ, junctional zone; L, labyrinth; D, decidua.

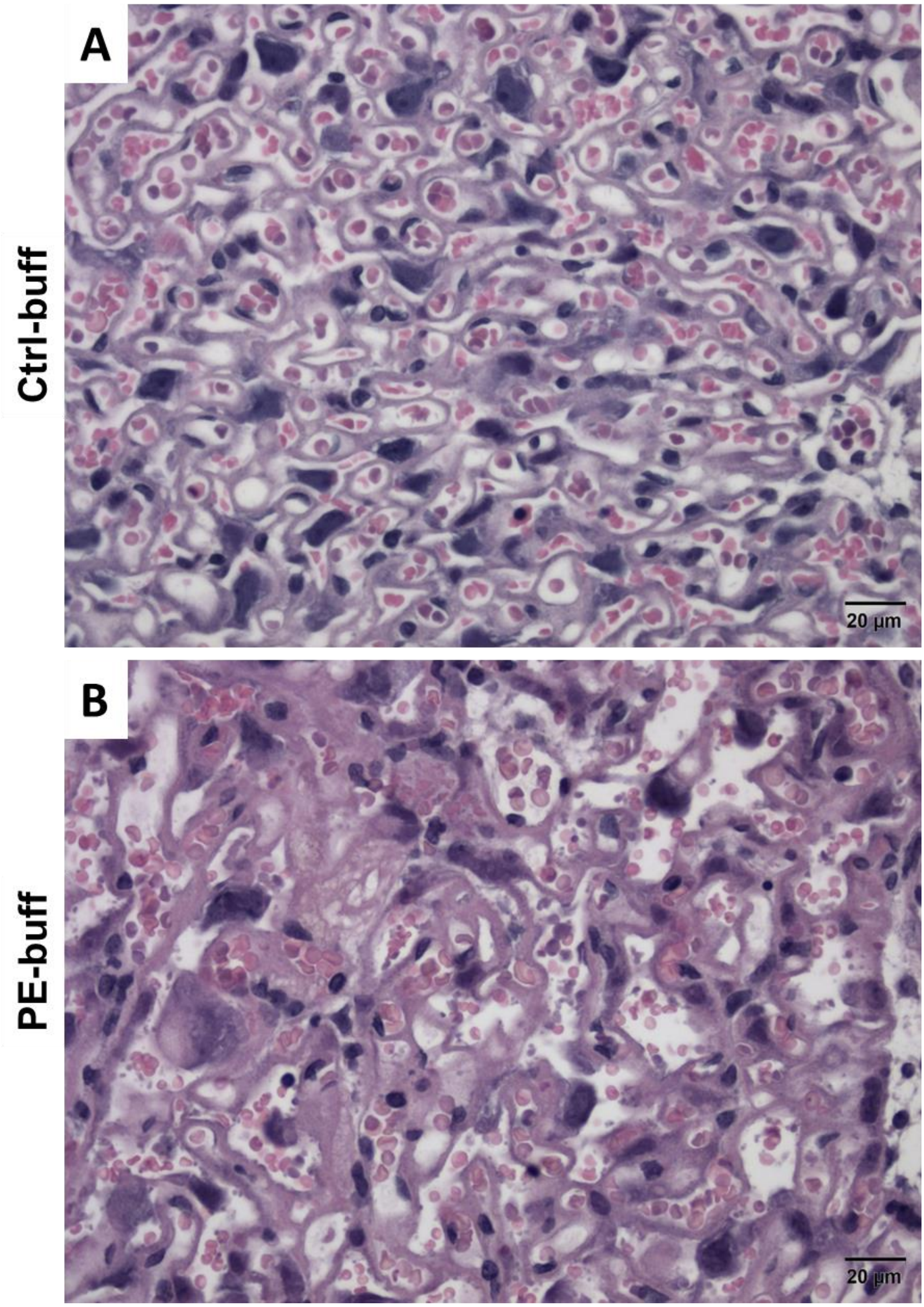

Figure S4. H&E analysis of placenta biopsies.

Representative images showing unclear structures with a fuzzy appearance in the labyrinth zone of the placenta from the PE-buff group (B) compared to the control (A-Ctrl-buff). Scale bar: 20µm.

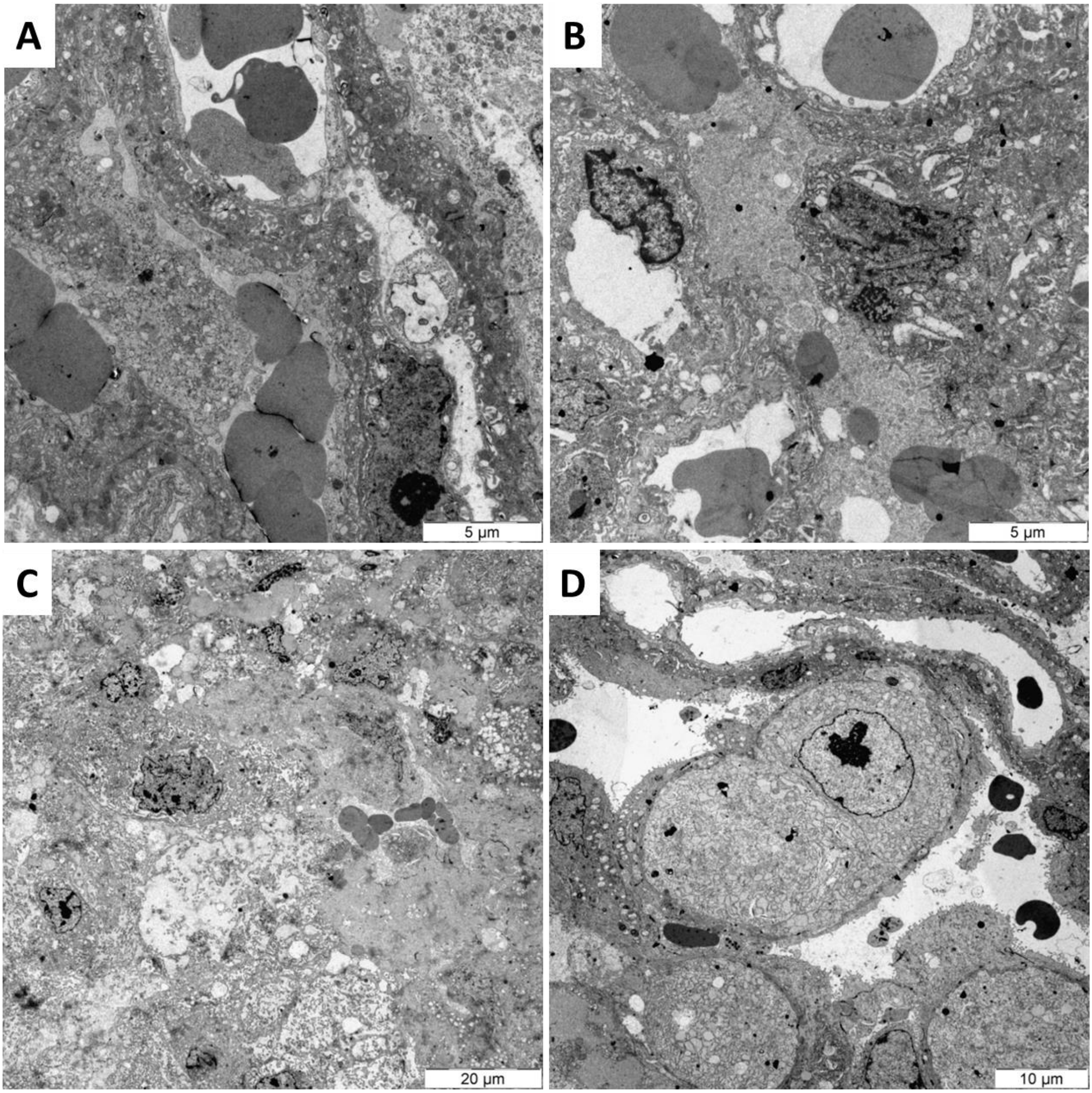

Figure S5. TEM analysis of placenta biopsies.

Representative images showing extended tissue damage in placenta from PE-buff (C) compared to the control groups (A-Ctrl-buff, B-Ctrl-A1M) and the PE-A1M placenta (D). Scale bar: 5µm (A, B), 20µm (C) and 10µm (D).

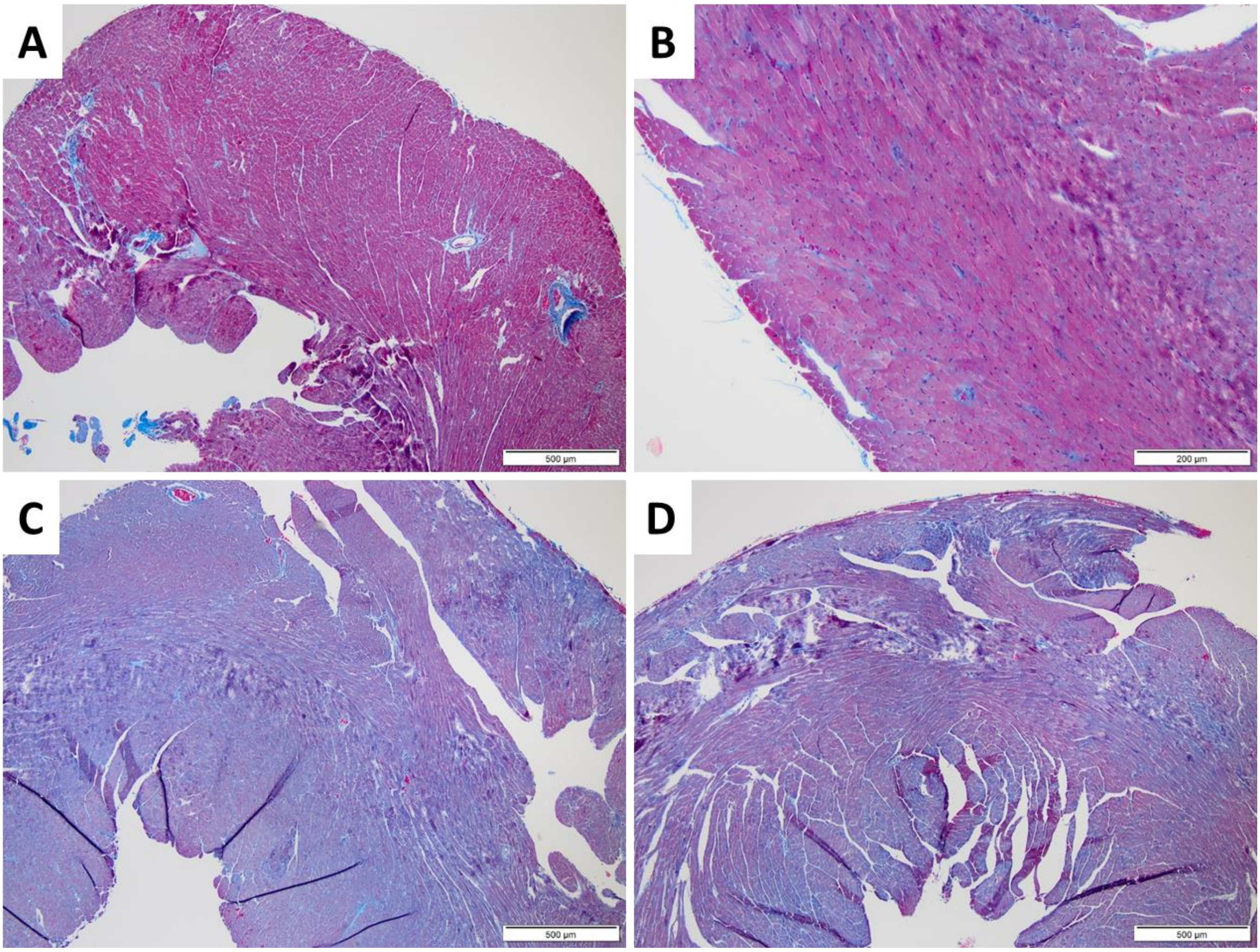

Figure S6. Masson's Trichrome staining of heart biopsies.

A more intense blue staining (Aniline blue specific for collagen) for (C) PE-buff and (D) PE-A1M compared to the two control groups (A) Ctrl-buff and (B) Ctrl-A1M. Scale bar: 500µm (A, C, D) and 200µm (B).

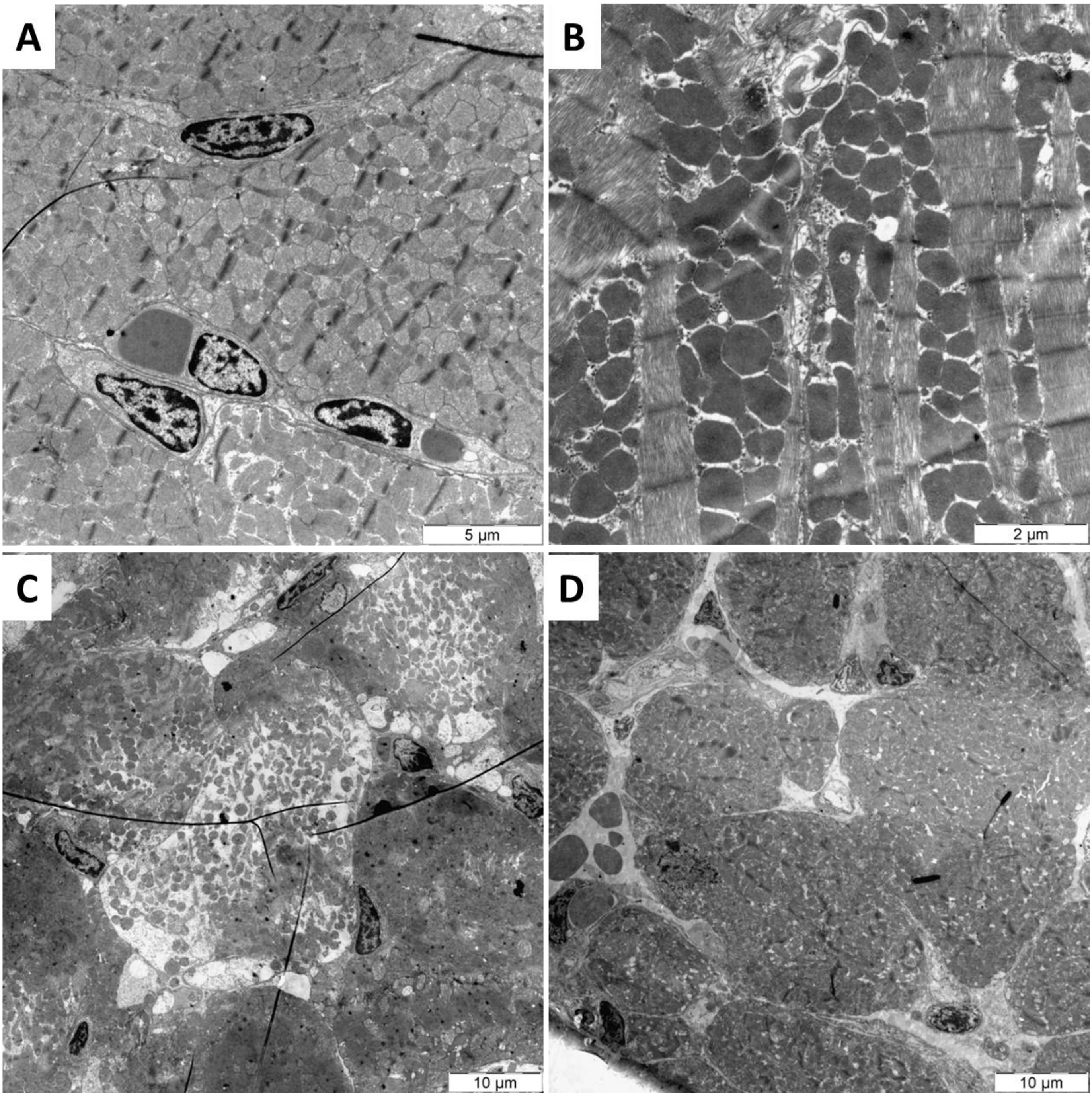

Figure S7. TEM analysis of heart biopsies.

Representative images showing extended tissue damage in heart from PE-buff (C) with loss of striated appearance and tissue structure, compared to the control groups (A-Ctrl-buff, B-Ctrl-A1M). Treatment with rA1M restores some of the structure and the striated appearance (D). Scale bar: 5µm (A), 2µm (B), 10µm (C, D).

Table S1. Cardiac magnetic resonance imaging in pregnant mice at 17·5 dpc.

MRI	Control (n=4)	Preeclampsia (n=4)
Heart rate (beats per minute)	445 (435 - 476)	520 (435 - 555)
LV-EDV (µl)	62 (60 - 63)	65 (61 - 67)
LV-ESV (µl)	24 (22 - 25)	26 (22 - 28)
LV-EF (%)	62 (60 - 63)	60 (54 - 67)
LV-CO (ml/min)	17 (16 - 18)	19 (18 - 22)*
LV mass (mg)	81 (74 - 86)	88 (82 - 93)
RV-EDV (µl)	54 (52 - 59)	58 (53 - 65)
RV-ESV (µl)	21 (18 - 22)	21 (18 - 27)
RV-EF (%)	62 (60 – 66)	62 (59 - 70)

Preeclampsia vs control: *p=0.029. Shown is median (range).

LV, left ventricular; EDV, end-diastolic volume; ESV, end-systolic volume; EF, ejection fraction; CO, cardiac output; RV, right ventricular.

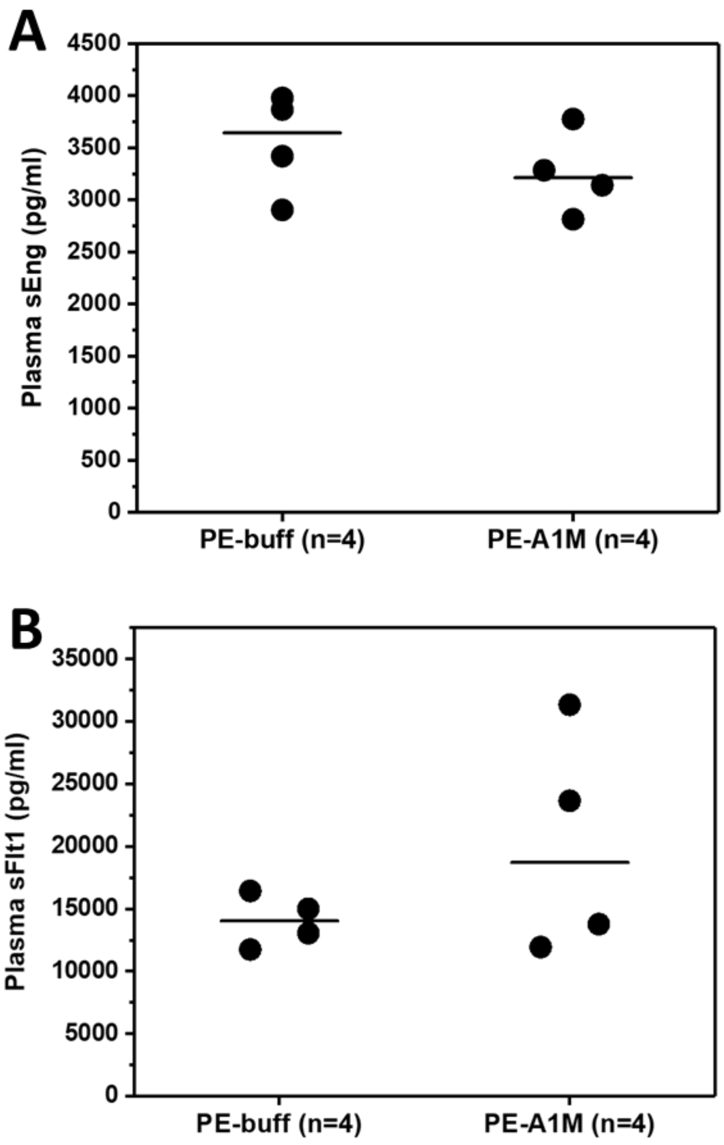

Figure S8. Plasma levels of sEng and sFlt1 is not reduced by rA1M treatment.

(A) No significant difference in plasma levels of sEng between PE-buff and PE-A1M groups.

(B) No significant difference in plasma levels of sFlt1 between PE-buff and PE-A1M groups.

The line represents the median and n=number of females analysed.

Table S2. Number of females included

	Paris	Lund
**Control-buff**	4	5
**Control-A1M**	4	2
**PE-buff**	5	6
**PE-A1M**	5	3
**Ctrl-MRI**	-	4
**PE-MRI**	-	4
**Non-pregnant**	-	5
